# Obesity May Provide Pro-ILC3 Development Inflammatory Environment in Asthmatic Children

**DOI:** 10.1155/2018/1628620

**Published:** 2018-12-06

**Authors:** Yumin Wu, Jiawei Yue, Juncheng Wu, Wei Zhou, Dapeng Li, Kai Ding, Prince Amoah Barnie, Xu Xu, Huaxi Xu, Weifeng Shi

**Affiliations:** ^1^Department of Laboratory Medicine, The Third Affiliated Hospital of Soochow University, Changzhou 213003, China; ^2^Department of Orthopaedics, The Third Affiliated Hospital of Soochow University, Changzhou 213003, China; ^3^Department of Gastroenterology, Shanghai General Hospital, Shanghai Jiao Tong University School of Medicine, Shanghai 200080, China; ^4^Department of Orthopaedics, Affiliated Hospital of Jiangsu University, Zhenjiang, Jiangsu Province 212001, China; ^5^Department of Biomedical Science, School of Allied Health Sciences, University of Cape Coast, Ghana; ^6^Department of Immunology, School of Medicine, Jiangsu University, Zhenjiang, Jiangsu Province 212013, China

## Abstract

The prevalence of obesity in children has dramatically increased in the last few decades, and obesity has also emerged as an important risk factor for asthma. Innate mechanisms have been shown to be involved in both diseases, particularly through the recently described innate lymphoid cells (ILCs), in which ILC3s have been linked to obesity both in human and in murine models. The aim of this study was to explore whether being overweight in asthmatic children was associated with elevated circulating ILC3 or elevated messenger RNA (mRNA) levels of RORC, IL-17A, and IL-22. Our results showed significantly elevated ILC3 frequencies in overweight asthmatic children compared with nonoverweight controls based on the detection of Lin^+^CD127^+^IL-23R^+^ cells by flow cytometry. Moreover, elevated ILC3 frequencies positively correlated with the mRNA expression of RORC which has been identified as a transcription factor of ILC3s. The relative mRNA expression level of IL-17A was also upregulated in overweight compared to nonoverweight children, as was the relative mRNA level of IL-22. However, there were no correlations between ILC3 frequencies or the expressions of RORC, IL-17A, and IL-22 and asthma severity. These results suggested that childhood obesity is an independent factor that is associated with an elevated frequency of circulating ILC3s and higher expressions of RORC, IL-22, and IL-17A.

## 1. Introduction

Obesity not only affects adults by causing chronic diseases including arterial hypertension, type 2 diabetes, or coronary heart disease but also has emerged as an important risk factor for asthma [1–3]. As a consequence of the modern eating habits associated with a sedentary lifestyle, the prevalence of obesity worldwide has been increasing [4]. With the growing prevalence of obesity, a concomitant rise in the incidence of bronchial asthma has been observed in the last few years [5, 6]. Obesity is known to contribute to a low-grade chronic inflammation with higher numbers of mast cells, macrophages, T- and B-cells, and neutrophils in adipose tissue [7, 8]. But the underlying pathogenesis of obesity contributes to asthma and complex mutual interactions between immunity are not yet completely understood.

Asthma is a heterogeneous inflammatory disease, characterized by chronic inflammation of the airways, increased mucus production in the bronchioles, and airway hyperreactivity to various stimuli [9]. Asthma is such a common disease, and its prevalence has increased worldwide over the last decades, which estimate that asthma affects approximately 300 million individuals globally [10]. Several cell types involve in airway inflammation, adaptive immunity, and antigen-specific Th2 cells have been correlated with the severity of diseases for several decades for the production of Th2 cytokines [11]. However, although Th2 cells can explain many of the features of asthma, it has become very clear over the past 5 years that asthma has heterogeneous and complex traits much more than Th2 cells [12].

Recently, an emerging innate immunity family of non-T and non-B effector cells has been confirmed to play crucial roles in the resistance to pathogenic and nonpathogenic microorganisms and in tissue homeostasis, repair, and remodeling which are called ILCs [13–15]. Unlike adaptive immune cells, ILCs are antigen nonspecific which lack rearranged antigen-specific receptors and lack dendritic cell phenotypical markers and myeloid cell markers but react promptly to a wide range of innate signals. The ILC populations can be categorized into three groups and the nomenclature is based on helper T cell nomenclature: (1) group 1 ILCs (ILC1s), which can produce interferon g (IFN-*γ*) [16]; (2) group 2 ILCs (ILC2s), which produce type 2 cytokines, in particular IL-5 and IL-13 [17]; (3) group 3 ILCs (ILC3s), which produce IL-17 and/or IL-22 [18]; and regulatory ILCs (ILCreg), which produce IL-10 and TGF*β* [19]. Many studies highlight the role of ILC2s in asthma. As a counterpart of Th17 cells, ILC3s is also proved to participate in many diseases such as atherosclerosis [20] and inflammatory bowel disease (IBD) [21]; furthermore, there is growing evidence that ILC3s might also contribute to adiposity and metabolic changes [22]. Interestingly, ILC3s elevate in the lungs and livers of mice with a HFD-induced obesity compared to normal-weight mice [23]. More ILC3s were detected in adipose tissue of obese compared to normal-weight mice [23]. However, it remains unknown whether elevated ILC3s in overweight individuals link with obesity or are a consequence of associated chronic inflammatory diseases. In this study, we wanted to test the hypothesis that ILC3 frequency is elevated in the peripheral blood of overweight children with asthma.

## 2. Materials and Methods

### 2.1. Patients

The study was performed at the Third Affiliated Hospital of Soochow University (Changzhou, China) and reviewed and approved by the ethical committee of the Third Affiliated Hospital of Soochow University (Changzhou, China). Written informed consent was obtained from older children themselves and from the parents of the younger children. Fifty asthma children were recruited from our outpatient clinic who were diagnosed with mild to severe asthma based on commonly accepted clinical and laboratory guidelines. All the patients were untreated for their condition at the time of blood collection. When they were younger than 4 years or older than 17 years old, had atopic dermatitis, allergic rhinoconjunctivitis, or any autoimmune disease in their medical history, they were excluded from our study. In addition, children were also excluded when they had an acute infection during the previous 2 weeks, clinical symptoms of a current infection, current C-reactive protein elevation, or leukocytosis. Body mass index (BMI) was calculated as a continuous variable from their height and weight (kg/m^2^) given debates within the literature as to the appropriateness of WHO guidelines for classifying overweight and obesity in the Chinese population [24]. Children with a body mass index (BMI) of >90th percentile were included in our overweight group, and our nonoverweight control group included children with a BMI of <90th percentile.

### 2.2. Cell Preparation and Flow Cytometric Quantification

Peripheral venous blood samples were collected in heparin-containing tubes and peripheral blood mononuclear cells (PBMCs) were isolated by Ficoll-Hypaque density-gradient centrifugation (GE Healthcare, Tokyo, Japan). The PBMCs were divided into two equal aliquots; one was used for flow cytometric quantification immediately, and then 1 ml TRIzol (Invitrogen, USA) was added into the other aliquots and was cryopreserved at −80°C for extracting total RNA. For flow cytometric quantification, the cells were stained with fluorochrome-conjugated mAbs specific for the following phenotypic markers according to the manufacturer's instructions. These antibodies were used in this study: anti-lineage (CD2, CD3, CD14, CD16, CD19, CD56, and CD235a; eBioscience, San Diego, CA, USA), anti-IL-23R-PE (BioLegend), and anti-IL-2R*α*-APC-R700 (BD Biosciences). After incubation, the samples were washed with phosphate-buffered saline (PBS), and the pellets were resuspended in 250 *μ*l of PBS. As for the control, the appropriate isotype-matched antibodies were used for each staining. The labeled cells were quantified by the fluorescence-activated cell sorting (FACS) LSRFortessa cell analyzer (BD Biosciences). The FACS data were analyzed with Flowjo10 software (version 7.6.5; Tree Star, Ashland, OR, USA).

### 2.3. RNA Isolation, cDNA Transcription, and Real-Time PCR

An aliquot of the PBMCs was used for total RNA isolation with TRIzol reagent (Invitrogen, Carlsbad, CA) following the manufacturer's instructions, and RNA concentration was measured on a NanoDrop 2000c (Thermo Fisher Scientific, Nidderau, Germany) and was diluted by the addition of RNase free water (water, mol biograde DNase-, RNase-, and protease-free; TAKARA) into a concentration of 500 ng/*μ*l. Then, an equal amount of RNA was used for real-time RT-PCR analyses using the SYBR Green Premix EX Taq kit (TaKaRa, Otsu, Japan). Real-time PCR was performed on each RNA sample to determine the relative expression of CD45, RORC, IL-23, IL-22, and IL-17A mRNA and was normalized by *β*-actin as a housekeeping gene and calculated with the comparative threshold cycle (Ct) method. On the basis of GeneBank sequences, the primers used in this study were designed by Premier 5.0 software and synthesized by Shanghai Invitrogen. All sequences of primers are shown in Table 1. Each sample was analyzed in duplicate with the CFXA96 Cycler (Thermal).

### 2.4. Statistical Analysis

All statistical analyses were performed by using Prism 5 (Graph Pad Software, La Jolla, CA, USA). Data were expressed as mean ± SD. Statistical comparisons between groups were performed using Student's unpaired *t*-test. For correlation between two continuous variables, Spearman's test was used. Differences were statistically significant when the *p* values were 0.05 or less.

## 3. Results

### 3.1. Circulating Baseline Characteristics in Patients with Asthma

Fifty children between 4 and 17 years of age with asthma were included in this study: 20 were overweight and 30 were not overweight (the controls). The patients' peripheral blood baseline characteristics, separated by the severity of asthma, are shown in Table 2. The overweight children had a mean BMI percentile of 96.3 ± 2.72 in comparison to 37.7 ± 25.69 for the control group. The circulating characteristics of the eligible patients are summarized in Table 2. The male-to-female ratio was 22 : 28, the mean age of the overweight asthmatic group was 8.1 ± 2.73 years old, and the mean age of nonoverweight asthmatic group was 7.6 ± 2.15 years old. The male-to-female ratio in the overweight asthmatic group was significantly lower than that in the nonoverweight asthmatic group. In addition, the peripheral neutrophil counts in the overweight asthmatic group was significantly higher than those in the nonoverweight asthmatic group, while there were no differences in the peripheral eosinophil counts between males and females or between the overweight asthmatic and the nonoverweight asthmatic group.

### 3.2. ILC3s Are Significantly Increased in Patients with Asthma

**Figure 1:**
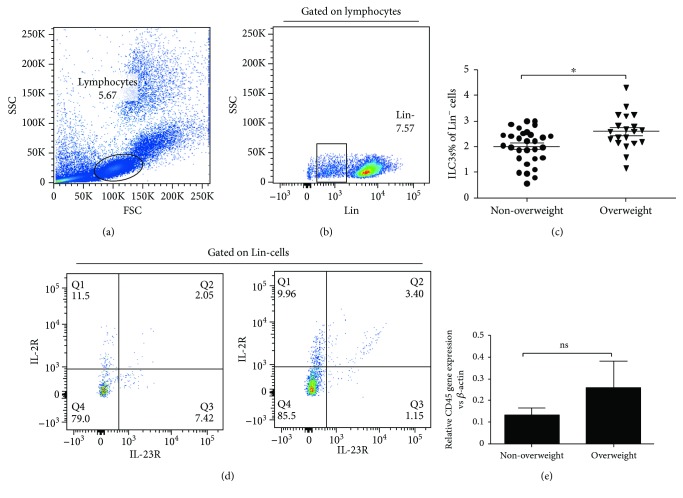
Enhanced ILC3 frequency in the PBMC from nonoverweight children and overweight children with asthma. The frequency of ILC3s in PBMC was analyzed by flow cytometry. (a–c) Representative diagrams of flow cytometry analysis for circulating ILC3s. (d) The frequency of ILC3s in PBMC from overweight children with asthma was significantly increased compared to nonoverweight children with asthma. (e) Representative diagrams of the mRNA level of CD45 from children with asthma. ^∗^*p* < 0 05; ns: not significant.

**Figure 2:**
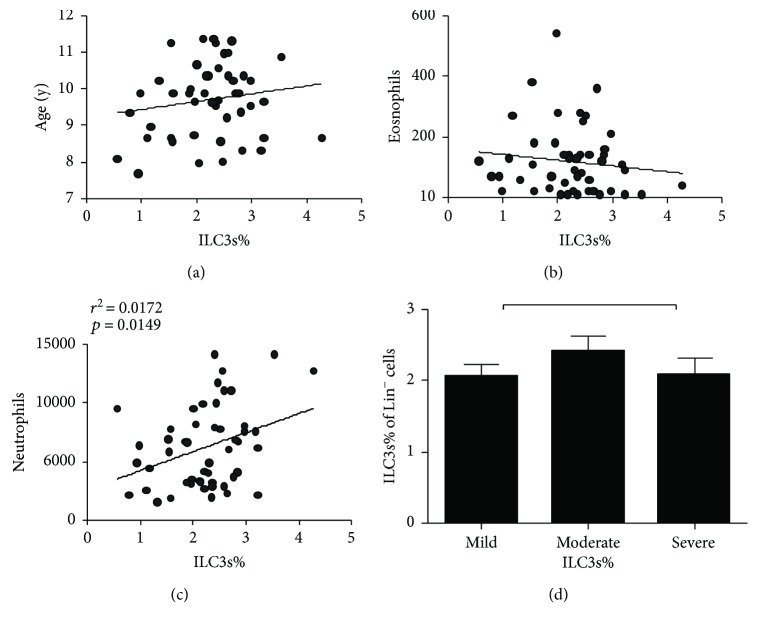
Correlation analysis between the proportion of ILC3s and the patients' age, peripheral neutrophil count, peripheral eosinophil count, or asthma severity. (a) The correlation of ILC3s% and the patients' age. (b) The correlation of ILC3s% and peripheral eosinophil count. (c) The correlation of ILC3s% and peripheral neutrophil count. (d) The correlation of ILC3s% and asthma severity. Data shown were represented as mean ± SD (all samples were measured in triplicate). ns means not significant.

Here, we examined the relative and absolute numbers of ILC3s in the peripheral blood of 50 asthmatics at diagnosis. The gating strategy for PBMCs is shown in Figure 1. In this study, the frequency of ILC3s was demonstrated by the proportion of Lin^+^CD127^+^IL-23R^+^ cells. The frequency of ILC3s was elevated in the peripheral blood of overweight children with asthma compared with that in the peripheral blood of nonoverweight children with asthma (Figures 1(a)–1(d)). We also detected the expression of CD45, an antigen expressed on all leukocytes, and our results showed a similar tendency consistent with the results for the ILC3s (Figure 1(e)). However, there were no significant differences between the frequency of ILC3s and the patients' age, peripheral eosinophil counts or asthma severity (Figures 2(a), 2(b), and 2(d)). Interestingly, the frequency of ILC3s was positively correlated with peripheral neutrophil counts (Figure 2(c)).

### 3.3. Inflammatory Cytokines Levels in PBMC Samples

**Figure 3:**
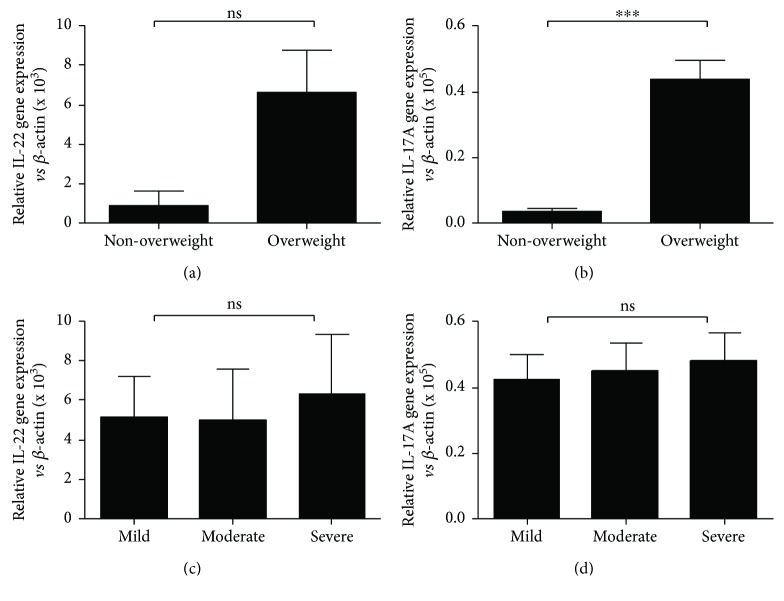
The PBMC levels of IL-17A and IL-22 in the study population. qRT-PCR analysis of IL-22 (a) and IL-17A (b) mRNA levels in PBMC from patients and correlation of IL-22 (c) and IL-17 (d) and asthma severity. Data shown were represented as mean ± SD (all samples were measured in triplicate). ^∗∗∗^*p* < 0 01 and ns means not significant.

**Figure 4:**
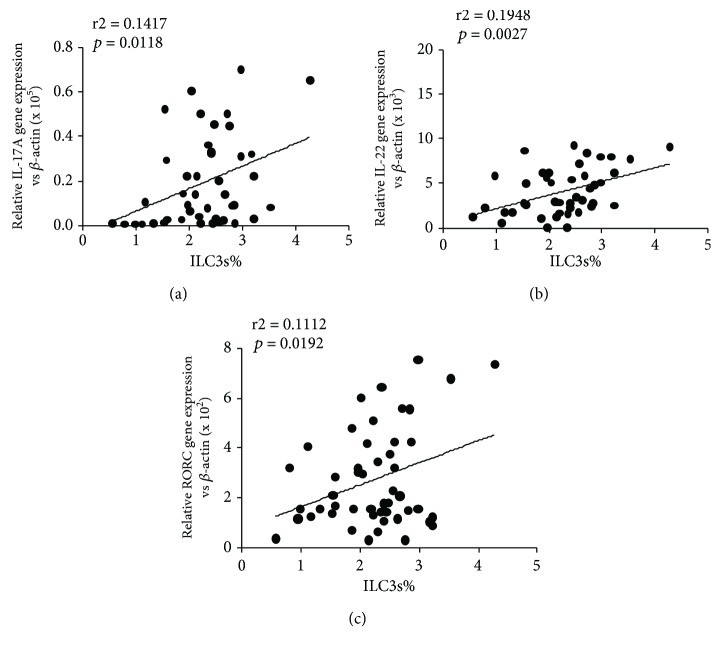
Correlations between IL-17A, IL-22, and RORC levels and ILC3s. (a) The correlation of ILC3s% and IL-17A mRNA expression (*p* = 0.0118, *r*^2^ = 0.1417). (b) The correlation of ILC3s% and IL-22 mRNA expression (*p* = 0.0027, *r*^2^ = 0.1498). (c) The correlation of ILC3s% and RORC mRNA expression (*p* = 0.0166, *r*^2^ = 0.1385).

Figure 3 displays the PBMC levels of two inflammatory cytokines in the study population. Without adjustment for confounders, IL-22 and IL-17A levels were higher in the overweight asthmatics (Figures 3(a) and 3(b)); however, there were no statistically significant differences in IL-22 and IL-17A levels linked to asthma severity (Figures 3(c) and 3(d)). Furthermore, our results showed linear correlations between IL-17A and IL-22 levels and ILC3s (Figures 4(a) and 4(b)).

### 3.4. Levels of RORC and IL-23 in the Serum of Asthmatics

**Figure 5:**
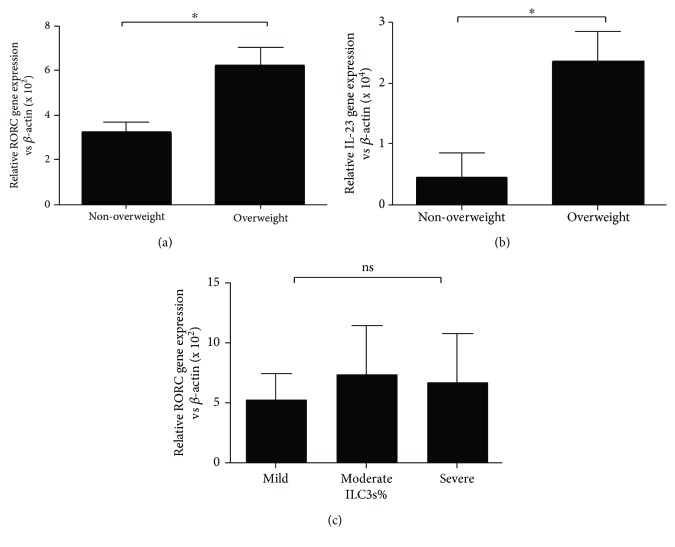
Levels of RORC and IL-23 in serum of asthmatic children. (a) The mRNA level of RORC in PBMC of asthmatic children. (b) The mRNA level of IL-23 in PBMC of asthmatic children. (c) The correlation of RORC mRNA expression and asthma severity. ^∗^*p* < 0 05; ns: not significant.

**Figure 6:**
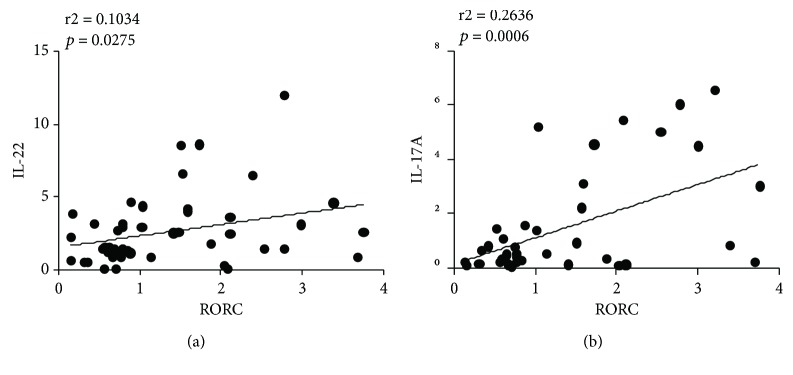
Correlation analysis between RORC and IL-22 and IL-17A mRNA levels. (a) The correlation of IL-22 and RORC mRNA expression (*p* = 0.0275, *r*^2^ = 0.1034) and (b) IL-17A and RORC mRNA expression (*p* = 0.0006, *r*^2^ = 0.2636) in asthmatic children; there was a positive correlation between them.

Because RORC is identified as a transcription factor of ILC3s and because IL-23 has been identified as an activating factor of ILC3s, we measured the mRNA levels of RORC and IL-23 in the PBMCs of asthmatic children. As Figure 5 shows, the expression levels of RORC and IL-23 were higher in the overweight asthmatic group than those in the nonoverweight asthmatic group (Figures 5(a) and 5(b), *p* < 0.05); however, no statistically significant difference between RORC level and asthma severity was found (Figure 5(c)). Significant differences between ILC3 percentages and RORC levels and between RORC levels and IL-17A and IL-22 levels were found (Figures 4(c), 6(a), and 6(b)).

## 4. Discussion

In this study, we observed that in patients with asthma, the frequency of circulating ILC3s was significantly increased in overweight asthmatic children compared to nonoverweight controls. In agreement with this finding, our data showed a significantly higher expression of IL-23, IL-17A, IL-22, and RORC-mRNA transcripts in PBMCs from overweight children than in PBMCs from nonoverweight children. Our data also showed statistically significant positive correlations between ILC3 frequency and IL-17A mRNA expression and peripheral neutrophil counts; however, there were no correlations between the frequency of circulating ILC3s, IL-23, IL-17A, IL-22, and RORC levels and asthma severity.

Obesity and asthma are often coassociated, and the association between obesity and asthma has been known for several years [25, 26]. The prevalence of asthma in obese subjects is higher than that in lean subjects, and these two diseases appear to affect each other. Kim et al. reported that severity of asthma increased with increasing BMI [27], and bariatric surgery induced weight loss, improved small airway function, and decreased systemic inflammation and number of mast cells in the airways [28]. In addition, several recent studies have identified the prevalence of metabolic dysregulation in obese children with asthma [29–31], but the specific mechanisms by which obesity causes asthma have not been defined until now. Steinberg et al. reported that mice with diet-induced obesity exhibit innate AHR and enhanced pulmonary inflammation [32]. Moreover, using a murine model of allergic asthma, Kim et al. found that obesity-associated asthma is facilitated by inflammation mediated by ILC3 cells [23]; however, the precise mechanisms that link obesity with airway inflammation have not been elucidated. Since obesity is linked to an increased risk of asthma and ILC3s are increased in patients with such conditions, we studied obese individuals with asthma.

The role of ILC3s in the development of asthma is still under investigation; researchers have examined the role of ILC3s in the development of asthma in mice and humans [23, 33]. When recombinant IL-17A is administered to the lungs of mice, airway inflammation and AHR are induced by the contraction of smooth muscle cells [34]. Moreover, IL-17A in the peripheral blood or sputum of patients with asthma is correlated with the severity of asthma [35, 36]. IL-17A is recognized as a characteristic cytokine secreted by Th17 cells; however, ILC3s can also produce IL-17A. In a mouse model of obesity, mice fed with a high-fat diet had significantly elevated numbers of ILC3-producing IL-17A in their lungs and developed AHR spontaneously [23]. Meanwhile, ILC3s were also observed in the bronchoalveolar lavage fluid of patients with asthma [23].

Since BMI standard values vary between various ethnicities, we used the appropriateness of WHO guidelines for classifying overweight in the Chinese population [24]. We collected the patients' peripheral blood baseline characteristics and separated them by the severity of asthma (Table 2), and then we detected the frequencies of ILC3s in the overweight and nonoverweight groups (Figures 1(a)–1(c)). Intriguingly, ILC3 frequencies were apparently higher in the overweight groups compared with the nonoverweight groups (Figure 1(a)), which may indicate that obesity provides an inflammatory environment that promotes ILC3 development. However, there was no correlation between the proportion of ILC3s and the patients' age, peripheral eosinophil count, or asthma severity (Figures 2(a), 2(b), and 2(d)).

ILC subsets are defined by characteristic expression patterns of surface antigens, characteristic cytokines, and master transcription factors. For instance, human ILC1s produce interferon-*γ* and T-beta [37], ILC2s produce IL-13 and RORA [38–40], and ILC3s produce IL-17A and RORC, which is orthologous to ROR*γ*t in mice [41]. RORC as a master transcription factor is necessary for the differentiation of CHLP into ILC3s and is important for the production of ILC3 effector cytokines in humans, and ILC3s are defined by their expression of RORC and characteristic cytokines IL-17A and IL-22 [41]. The expression levels of RORC, IL-17A, and IL-22 transcripts were investigated in unsorted PBMCs (Figures 3(a), 3(b), and 5(a)), which may include Th17 cells, another source of IL-17A, IL-22, and RORC expression. Our results show linear correlations between RORC, IL-17A, and IL-22 levels and ILC3s (Figure 4).

Multiple different staining protocols have been developed for ILC3s; ILC3s express retinoic acid receptor- (RAR-) related orphan receptor (ROR*γ*t), respond to IL-1*β* and IL-23 or to danger/pathogen signals [42], and produce IL-17A and/or IL-22. In the present study, the staining protocol defined ILC3s as Lin^−^IL-2R*α*^+^IL-23R^+^, which is a common definition in the flow cytometric assessment of ILC3s [43]. These Lin^−^IL-2R*α*^+^IL-23R^+^ cells might include two fraction of cells, namely, NKp^+^44 ILC3s and NKp^−^44 ILC3s; however, according to previous work, NKp^+^44 ILC3s mainly secrete IL-17A and NKp^−^44 ILC3s mainly secrete IL-22. Thus, we measured the levels of these two cytokines, and our results also showed linear correlations between ILC3 levels and IL-17A and IL-22 levels (Figures 4(a) and 4(b)). Meanwhile, because RORC is identified as a transcription factor of ILC3s, our results also show linear correlations between RORC and IL-22 and IL-17A (Figure 6).

Obesity does not only interfere with metabolic pathways but also interfere with immunological pathways [44]. Elevated ILC3s levels have been detected in the lungs and livers of mice with HFD-induced obesity compared to normal-weight mice, and more ILC3s have been detected in the adipose tissue of obese mice than in normal-weight mice [23]. Our results also showed an elevated ILC3 levels in obese asthmatic patients compared with nonobese asthmatic patients. Moreover, in humans, being overweight is associated with higher amounts of circulating neutrophils attracted by IL-17A [45, 46], which is in line with our findings that higher amounts of circulating neutrophils were present in obese asthmatic patients and that this was associated with higher IL-17A levels and ILC3 frequencies (Figure 2(c)).

## Figures and Tables

**Table 1:** The primer sequences for RT-PCR.

Gene	Sequence (5′-3′)	Accession
CD45	Fwd: GTGAGGCGTCTGTACTGATG	NM_002838
	Rev: ACGGCTGACTTCCAGATATG	
RORC	Fwd: CCGAGATGCTGTCAAGTTCG G	NM_001001523.1
	Rev: GTTCCTGTTGCTGCTGTTGC	
IL-23	Fwd: ACAGAGAGAATCAGGCTCA	NM_016584.2
	Rev: GGTACACAGGGTGATCA	
IL-22	Fwd: CAGGCTCAGCAACAGGCTAA	NM_020525.4
	Rev: TGATCTCTCCACTCTCTCCAAGC	
IL-17A	Fwd: CCTGGAGGCCATAGTGAAGG	NM_002190.2
	Rev: TTCCGGTTATGGATGTTCAGG	
*β*-Actin	Fwd: TGGCACCCAGCACAATGAA	XM_005249820.1
	Rev: CTAAGTCATAGTCCGCCTAGAAGCA	

**Table 2:** Characteristics of the study population.

Characteristic	Overweight	Controls	*p*
Number	20	30	ns
Age (years) (mean ± SD)	8.1 ± 2.73	7.6 ± 2.15	ns
Sex, female/male	13/7	11/19	ns
Body mass index (kg/cm^2^)	96.3 ± 2.72	37.7 ± 25.69	<0.05
Peripheral leukocytes (/*μ*l)	7.2 ± 1.21	5.6 ± 1.71	<0.05
Peripheral eosinophils (/*μ*l)	232.4 ± 210.95	237.4 ± 197.39	ns
Peripheral neutrophils (/*μ*l)	4255.7 ± 1728.45	3254.2 ± 878.65	<0.05

## Data Availability

The data used to support the findings of this study are included within the article.
